# Ultrasensitive single-nucleotide polymorphism detection using target-recycled ligation, strand displacement and enzymatic amplification†
†Electronic supplementary information (ESI) available: The preparation, modification and magnetic retrieval of MNPs, optimisation of the Cap-MNP probe and experimental procedures and the SNP discrimination at low target abundance (5 fmol). See DOI: 10.1039/c3nr01010d



**DOI:** 10.1039/c3nr01010d

**Published:** 2013-05-02

**Authors:** Yue Zhang, Yuan Guo, Philip Quirke, Dejian Zhou

**Affiliations:** a School of Chemistry and Astbury Centre for Structural Molecular Biology , University of Leeds , Leeds LS2 9JT , UK . Email: y.guo@leeds.ac.uk ; Email: d.zhou@leeds.ac.uk ; Fax: +44 (0)113 3436565; b Section of Pathology and Tumour Biology , Leeds Institute of Molecular Medicine , University of Leeds , Wellcome Trust Brenner Building, St James's University Hospital , Leeds LS9 7TF , UK

## Abstract

We report herein the development of a highly sensitive and selective approach for label-free DNA detection by combining target-recycled ligation (TRL), magnetic nanoparticle assisted target capture/separation, and efficient enzymatic amplification. We show that our approach can detect as little as 30 amol (600 fM in 50 μL) of unlabelled single-stranded DNA targets and offer an exquisitely high discrimination ratio (up to >380 fold with background correction) between a perfect-match cancer mutant and its single-base mismatch (wild-type) DNA target. Furthermore, it can quantitate the rare cancer mutant (KRAS codon 12) in a large excess of coexisting wild-type DNAs down to 0.75%. This sensor appears to be well-suited for sensitive SNP detection and a wide range of DNA mutation based diagnostic applications.

## Abstract 2

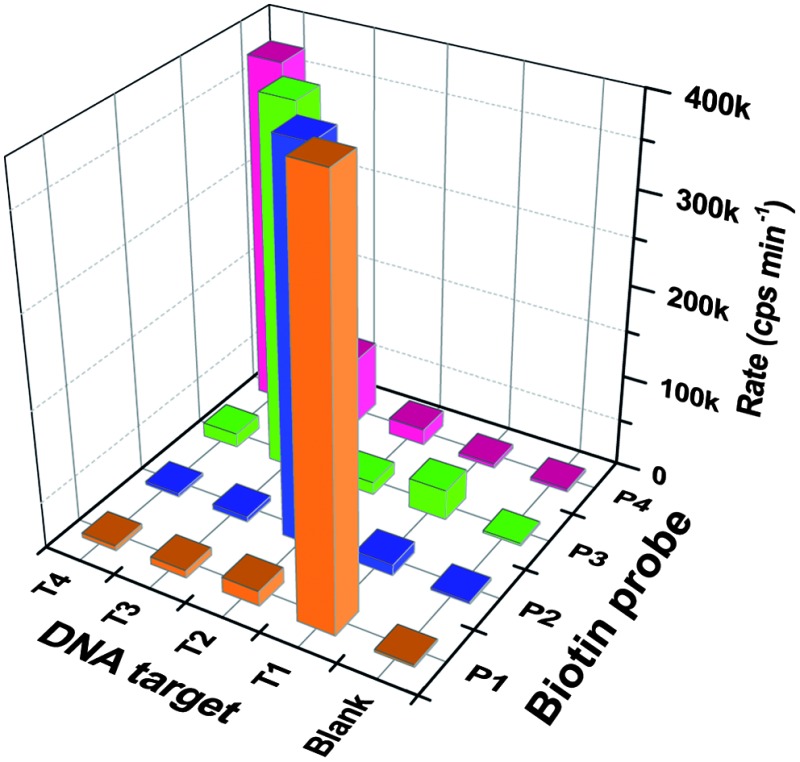
A novel, general, ultrasensitive DNA sensor offering fM sensitivity, exquisite SNP discrimination and accurate rare cancer mutant quantitation is developed.

Single-point mutations (SPMs) or single-nucleotide polymorphisms (SNPs) in genetics are associated with numerous important human diseases, such as cancer, diabetes, vascular diseases, and some forms of mental illness, *etc.* Therefore, the ability of sensitive detection of specific SPMs and SNPs in particular genes has a considerable value in disease diagnosis, prediction of patients' responses to treatments, risk of relapse of diseases and outcomes.^1–5^ However, the inherent small difference in thermodynamic stability from a single-base mismatch between the perfect-match and its SNP targets makes it challenging to achieve both high SNP discrimination and sensitivity. Over the past decade, several different approaches capable of SNP discrimination have been reported, such as high resolution DNA melting analysis (∼μM level sensitivity),^6–10^ single molecule fluorescence (*via* different annealing and melting kinetics between the perfect- and SNP-targets);^11^ molecular beacons,^12–14^ hybridization chain reactions,^15^ surface enhanced Raman scattering^16^ and electrochemical chemical detection.^17^ Despite such developments, most methods reported so far have displayed relatively low SNP discrimination ratios (DRs, *ca.* <20 fold) and/or limited sensitivity, which can also suffer from poor fidelity when analyzing samples with a large excess of DNA contaminants.

More recently, several enzyme based approaches have been developed to improve SNP detection and discrimination. For example, nicking endonucleases^18^ and restriction enzymes^3^ have been used to discriminate SNPs from perfect-match targets *via* specific enzyme restriction sites. Nevertheless, these approaches are only suitable for detection of SNPs containing the enzyme recognition sequences, limiting their scope of application. Since enzymes, such as S1 nuclease, endonuclease (I, III and IV) and λ exonuclease, are suitable for all DNA sequences, with delicate designs, they can be used as a general method for SPM/SNP detection. However, the poor SNP discriminating ability of these enzymes means that they have to be combined with other more specific enzymes to achieve the desired SNP discrimination, such as the combined use of endonuclease IV and λ exonuclease in a recent SNP detection system.^19^ DNA polymerase, widely used in polymerase chain reactions (PCRs) for DNA target amplification, possesses high specificity and is suitable for SNP detection.^17,20^

On the other hand, ligation reactions based on Taq DNA ligase are general, and can be applied to any target of interest. Moreover, it has high selectivity in ligating two nicked DNA strands hybridised to a full-complementary DNA template over those having a single-base mismatch at the nicking site. It is therefore well-suited for sensitive SNP and point-mutation detections.^21,22^ Indeed, a few sensitive, specific assays that may be suitable for genotyping and point-mutation detection have been realized with the ligase-based approaches,^22–27^ such as the combination of the ligation chain reaction (LCR) and conjugate polymer based FRET (Förster resonance energy transfer),^23^ and gold nanoparticle based colorimetric assay^24^ for sensitive SNP detection. The ligation reaction has also been combined with other signal amplification strategies, *e.g.* rolling circle amplification,^25–27^ and polymerase mediated target displacement,^28^ to further enhance sensitivity. Despite these strategies, the development of simple, sensitive diagnostic assays suitable for rapid detection of SNPs and point mutations associated with specific diseases are highly valuable to clinicians. Herein, we report the development of a novel, general and highly sensitive approach for specific, label-free detection of SNPs using the KRAS somatic mutations (codons 12/13) that are widely found in several human cancers (*e.g.* colorectal, pancreas, ductal and lung) as the model cancer DNA targets. We show that this sensor can detect as little as 30 amol of unlabelled DNA targets, and can offer high discrimination, up to >380 fold after background correction, between the full-match and the SNP targets. It can quantitate the KRAS cancer SNP mutant in a large excess of coexisting wild-type DNA targets down to 0.75% level (*e.g.* <1% of the DNA targets being the SNP cancer mutant).

## Results and discussion

**Scheme 1:**
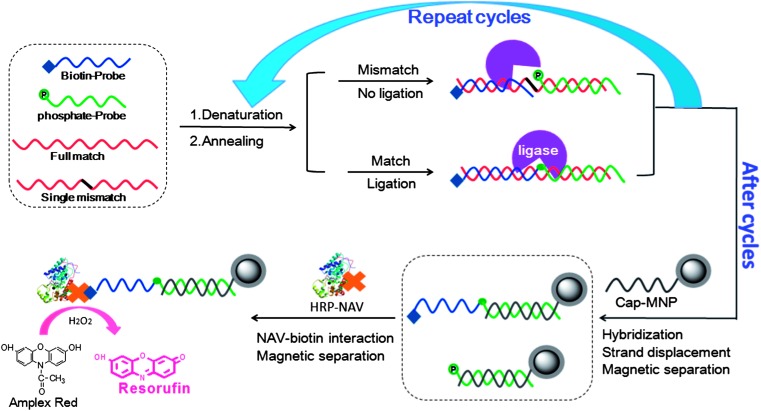
Schematic illustration of our strategy for specific SNP DNA detection. A biotin-probe (blue) and a phosphate-probe (green) are hybridized to each half of a complementary DNA target, which are then ligated by the Taq DNA ligase if the sequences between the probes and target are fully complementary, but not for those having a single-base mismatch (*ca.* SNP) at the nicking site. The system is then subjected to multiple cycles of denaturation, annealing and ligation, where each full-match template produces a ligated product in each cycle. A capture-DNA modified magnetic nanoparticle (note that hundreds of capture-DNA strands are linked to each MNP, only one is shown here for simplicity) was added to capture the ligated products, and followed by magnetic separation. Finally, a neutravidin conjugated horseradish peroxidase (NAV-HRP) is bound to the MNP, allowing for sensitive detection of the full-match DNA target *via* the HRP catalysed enzymatic assay.

Scheme 1 shows our approach schematically. Two short single-stranded (ss) DNA probes, each complementary to one half of the DNA target, are designed. The two probes are modified with a 5′-biotin (blue, for signal amplification) and 5′-phosphate (green, for magnetic capture of ligated product, see below), respectively. First, the two probes are hybridised to a ssDNA target, forming a nicked double-stranded (ds) DNA at annealing temperature (45 °C). The two probes hybridised to a full-complementary DNA target are subsequently ligated by the Taq DNA ligase at 45 °C, producing a covalently ligated DNA product with a terminal biotin (the ligated product). A single-base mismatch in the probe/SNP target duplex at the nick site can prevent such ligation, so the two probes remain unlinked. Upon heating to the denaturation temperature (95 °C), the DNA targets are dehybridised (released) from the ligated products (or unligated probes), which are subsequently used to template the ligation of a 2^nd^ pair of biotin-/phosphate-probes as the temperature is reduced to 45 °C. In this way, the DNA target is recycled, and hence this process is named as target-recycled ligation, TRL. Excess biotin-/phosphate-probes over the target DNA are used to minimize the re-hybridisation of the target to the ligated products. The process of repeating the denaturation, annealing and ligation thus recycles the DNA target, producing a ligated product during each cycle, leading to accumulation of the ligated products as the number of thermal cycles is increased.

After 5–30 thermal cycles, a capture-DNA (with a sequence complementary to the phosphate-probe section and in large excess) modified magnetic nanoparticle (Cap-MNP, see ESI† for details) is then added to capture the free ligated products *via* hybridization. For ligated products pre-hybridised to the DNA targets, the capture-DNAs may displace the DNA targets *via* a toehold mediated strand displacement. A large excess of the Cap-MNPs over the target DNA is used here (>100 fold) to ensure efficient target capture and strand displacement. After that, a magnetic separation followed by several rounds of washing is applied to separate the MNP-captured ligated products from unbound free species. The MNPs (with the captured products) are subsequently treated with a neutravidin conjugated horseradish peroxidase (NAV-HRP), converting each captured DNA product (with a terminal biotin) into a HRP enzyme *via* the strong, biospecific biotin–NAV interaction.^29,30^ This allows for sensitive detection of the ligated product (from the templated ligation by the full-match DNA target) *via* the efficient HRP catalysed conversion of a non-fluorescent substrate, Amplex red, into a strongly fluorescent product, resorufin,^29,31^ for real-time fluorescence monitoring on a conventional 96-well fluorescence plate reader. An advantage here is the general applicability: it can target any DNA of interest by simply changing the sequences of the phosphate-/biotin-probe pair. In practice, a common phosphate-probe and a specific biotin-probe (P2 or P3) are employed to detect each of the two KRAS mutants (T2 or T3, see Table 1) that are associated with human colorectal cancer.

**Table 1:** The DNA sequences and their abbreviations used in this study. The single base mutation between the wild-type and SNP targets is shown in italics

DNA code	DNA sequences
Phosphate-probe	PO_4_^–^-5′-T GGC GTA GGC AAG AGT ACG ACA-3′
Biotin-probe 1 (P1)	Biotin-5′-TTT TTT GTG GTA GTT GGA GCT G*G*-3′
Biotin-probe 2 (P2)	Biotin-5′-TTT TTT GTG GTA GTT GGA GCT G*A*-3′
Biotin-probe 3 (P3)	Biotin-5′-TTT TTT GTG GTA GTT GGA GCT G*T*-3′
Biotin-probe 4 (P4)	Biotin-5′-TTT TTT GTG GTA GTT GGA GCT G*C*-3′
Wild-type target (T1)	3′-CAC CAT CAA CCT CGA C*C*A CCG CAT CCG TTC TCA-5′
Mutant target 2 (T2)	3′-CAC CAT CAA CCT CGA C*T*A CCG CAT CCG TTC TCA-5′
Mutant target 3 (T3)	3′-CAC CAT CAA CCT CGA C*A*A CCG CAT CCG TTC TCA-5′
Mutant target 4 (T4)	3′-CAC CAT CAA CCT CGA C*G*A CCG CAT CCG TTC TCA-5′
Cap-DNA	3′-A CCG CAT CCG TTC TCA TGC TGT TTT TTT TTT-5′-C_6_SH

The stringent matching requirement of the Taq DNA ligase for ligating the two probes at the nick site^21,22^ enables this approach to be highly specific for the perfect-match over the SNP target(s). To separate the ligated products from other species after ligation, gel electrophoresis is commonly used, but it has poor sensitivity, often at the μM level.^32^ Alternative approaches using a solid support to capture and separate the ligated products can suffer from low hybridization efficiency and slow kinetics, limiting their sensitivity.^33^ MNPs are highly attractive here because of their tiny particle sizes and super-paramagnetic properties, allowing the formation of a stable, uniform dispersion in the media for rapid, homogeneous target capture without an applied magnetic field, while still being readily retrievable upon applying an external magnetic field (see ESI, Fig. S1†).^27,28,34–36^ Here, Cap-MNP is employed to capture the ligated products *via* strand displacement, where a ssDNA hybridises to a short complementary toehold first, leading to progressive displacement of the pre-hybridised strands.^37^ Its kinetics can be regulated by tuning the length of toeholds. Strand displacement is widely used in designing DNA nanomachines,^38^ logic circuits,^39^ hybridization chain reactions,^40^ DNA-templated syntheses,^41^ as well as DNA detection and point-mutation discrimination,^42–45^ but is rarely used for DNA capture and separation. In our design, a large excess of the Cap-MNPs (100–100 000 fold excess over the full-match target) is used here to hybridise the overhang toeholds in phosphate-probe/ligated product(s) and to displace pre-hybridised target strands. Moreover, the capture and displacement efficiency is also benefitted from the enhanced affinity of the Cap-MNPs for the ligated products *via* polyvalent binding similar to those observed for multivalent DNA–gold nanoparticle (GNP) conjugates, where 2 orders of magnitude higher binding affinity for the same DNA target has been reported for multivalent DNA–GNP conjugates over the corresponding doubled-stranded DNA in solution.^46,47^

### Detection of a perfectly matched target

Prior to using the Cap-MNP in target DNA detection, a series of experiments have been carried out to optimise the Cap-MNP probe and assay procedures to maximise the signal arising from the DNA target while minimising the background. These experiments included the amount of the Cap-MNP used in target capture (see ESI, Fig. S2A,† with 20 μg of Cap-MNP being found to be optimum), the capture-DNA loading on the MNP (ESI, Fig. S2B,† with 0.5 nmol (capture-DNA) per mg (MNP) being found to be optimum), the blockage of the Cap-MNP surface by 2-mercaptoethanol and BSA (see ESI, Fig. S2C,† this is key to achieving a high absolute signal-to-background ratio, SBR, by minimising the non-specific adsorption of NAV-HRP on the MNP surface), as well as the number of thermal cycles on the specific DNA signal (see ESI, Fig. S2D,† with 30 thermal cycles giving the highest specific signal). These optimised conditions were subsequently used for all of the specific target DNA detection experiments.

**Fig. 1:**
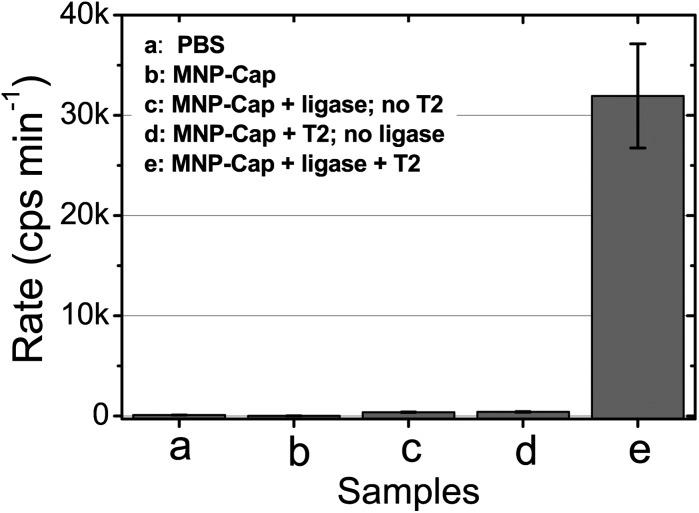
Fluorescence response (rate of increase, cps min^–1^) for different samples after identical thermal cycle treatments. All samples contain the same amount of Amplex red (2 μM) and H_2_O_2_ (2 μM). The samples are (a) PBS, (b) Cap-MNP in PBS, (c) Cap-MNP + ligase but no T2, (d) Cap-MNP + T2 but no Taq ligase, and (e) Cap-MNP + T2 + Taq DNA ligase.

After such optimisation experiments, we used our approach first to detect one of the KRAS cancer mutants (T2, see Table 1 for details) as the perfect-match target using the biotin-probe 2 (P2) and phosphate-probe. Taq DNA ligase was used to ligate the two probes templated by T2 (25 fmol). The result was compared against a set of control experiments (Fig. 1).

Fig. 1 reveals that the rate of fluorescence increase for the sample containing both the T2 and ligase (e) is much greater than that of the controls either containing no ligase (d, 79 (±12) fold) or T2 (c, 87 (±13) fold). This result clearly demonstrates that the fluorescence response observed herein is due to the T2 templated ligation only, with almost no contribution from non-specific adsorption of the NAV-HRP. The negligible false positive for controls without either the ligase or T2 (c or d) confirms the success and excellent specificity of our approach for target DNA detection.

**Fig. 2:**
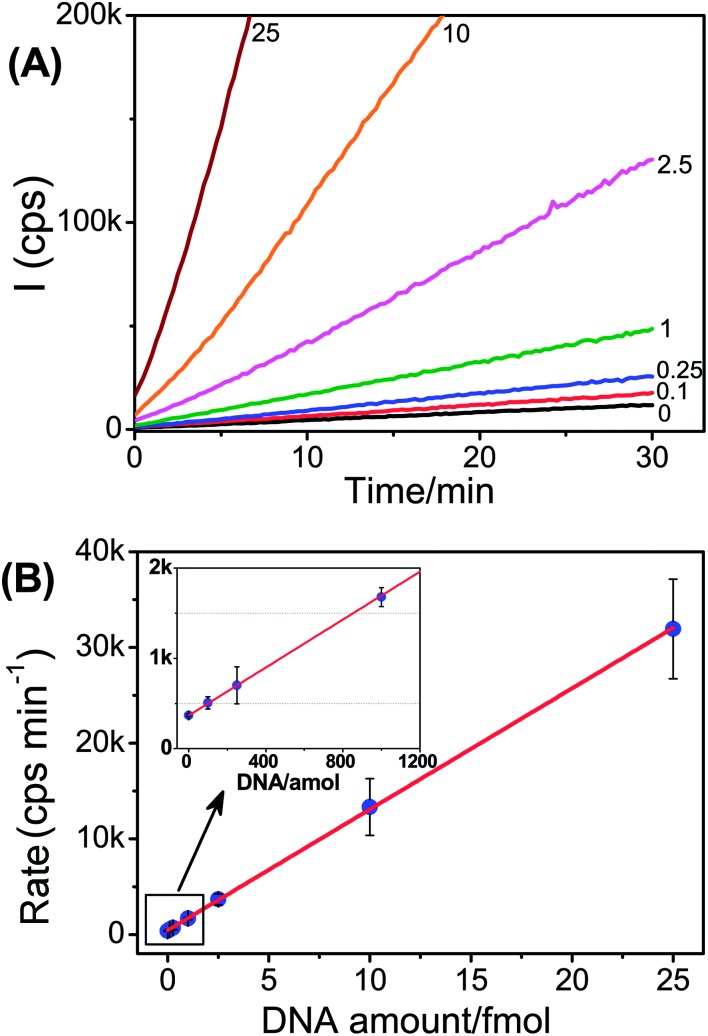
(A) Real time-dependent fluorescence responses for samples containing different amounts (0–25 fmol, the amounts are indicated on each curve) of the full-match DNA target, T2. (B) The corresponding correlation between the rate of fluorescence increase and amounts of the T2 target, fitted to a linear function (*y* = 368.8 + 65.2*x*, *R* ^2^ = 0.9997). Inset: amplified region over the 0–1200 amol T2 range.

Fig. 2A shows the time-dependent evolution of the fluorescence intensity at different amounts of the perfect-match target, T2, which clearly shows that the rate of resorufin production increases with the increasing amount of the target DNA. The relationship between the target DNA amount and the rate of fluorescence increase is found to be highly linear over the 0–25 fmol range (*R* ^2^ = 0.9997), suggesting that this approach can offer excellent target DNA quantitation accuracy. The limit of detection (LOD) based on three times the standard deviation at 0 T2/slope of calibration (*e.g.* 3*σ*/slope) is estimated as 30 amol (600 fM in 50 μL).^48^ The LOD here is comparable to or better than several recently reported sensitive DNA assays (Table 2). We attribute this high sensitivity to the great signal amplification power of the enzyme (where each NAV-HRP can turnover several hundred thousand non-fluorescent Amplex red substrates into strongly fluorescent resorufin products over a 30 min assay period),^29^ efficient target capture and magnetic separation, and the careful optimisation of the Cap-MNP probe which lead to negligible nonspecific adsorption of the enzyme, and hence greatly reduced background (ESI,† Fig. 2). In addition, the magnetic capture and separation process were also carefully arranged (see the Experimental section): the Cap-MNP was added after the thermal cycles to avoid the aggregation of the MNPs at high temperatures. We found that MNP aggregation can lead to decreased target capture efficiency and significantly increased background.

**Table 2:** The discrimination ratios (DRs) of specific biotin-probes for their respective full-match over other single-base mismatch DNA targets. The data shown in the brackets are after background correction, error bars are the standard deviations of 3 parallel samples. DR is defined as the signal ratio of the full-match target, P*n*/T*n*, over their respective SNP targets, P*n*/T*m* (where *n* ≠ *m*)

Target (base 17)	Probe			
	P1 (G)	P2 (A)	P3 (T)	P4 (C)
T1 (C)	1 (1)	35 ± 4.1 (43 ± 5.1)	12 ± 0.4 (13 ± 0.4)	91 ± 6.2 (298 ± 20)
T2 (T)	53 ± 15 (70 ± 20)	1 (1)	30 ± 1.1 (36 ± 1.2)	21 ± 1.4 (25 ± 1.7)
T3 (A)	57 ± 16 (78 ± 22)	76 ± 8.9 (133 ± 16)	1 (1)	5.2 ± 0.4 (5.4 ± 0.4)
T4 (G)	96 ± 27 (176 ± 50)	121 ± 14 (383 ± 45)	27 ± 1.0 (32 ± 1.1)	1 (1)
Blank	212 ± 60 (—)	175 ± 21 (—)	162 ± 5.8 (—)	130 ± 9 (—)

### Mimicking DNA melting assay

Melting temperature (*T*_m_) of nucleic acids is the temperature at which half of the dsDNAs are dehybridised into single-stranded (ss) structures. Melting is typically considered as the dissociation of the two strands in a dsDNA into two ss states, where intermediate states are often ignored.^10^ In our approach, a pair of the biotin-/phosphate-probes are first hybridised into target DNA (mutant or wild-type), forming a probe/target duplex with a nick site, which are then ligated by the ligase to form a covalently linked product. At high annealing temperatures, few probes can form stable sandwiches with the target DNA, leading to a decrease of the ligated products. Therefore the ligation efficiency (enzymatic activity after NAV-HRP binding) is a useful indication of the probe/target sandwich formation, which may be controlled by the annealing/ligation temperature.

**Fig. 3:**
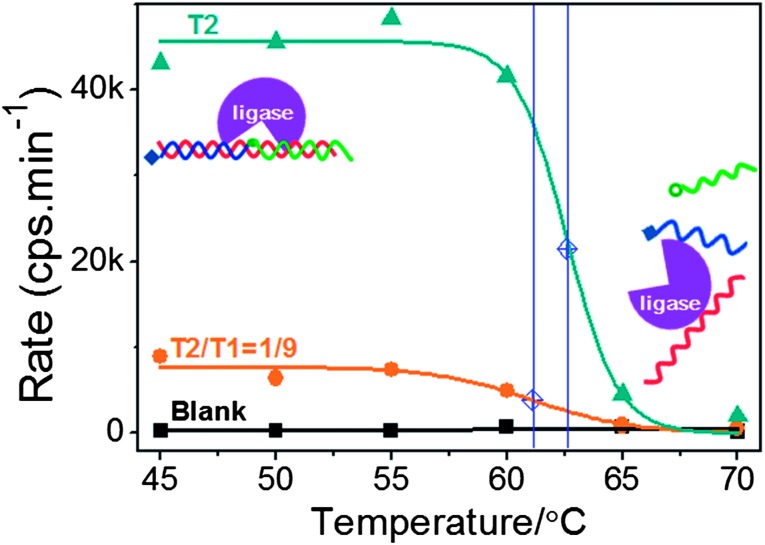
Relationship between the rate of fluorescence increase and ligation temperatures. According to the fitted melting curves, *T*_m_s were yielded and are indicated by blue solid lines crossing blue diamonds.

The effect of annealing temperatures on the detection of the full-match target T2 using the phosphate-/biotin-probe 2 was tested. The signal obtained from the SNP target only (*e.g.* T1) was found to be indistinguishable from the background (no target), due to the high specificity of our assay. Therefore two samples with the identical amount of total DNA (100 fmol), one containing the T2 only and the other being a T2–T1 mixture (T2 : T1 molar ratio = 1 : 9), were studied and the results are shown in Fig. 3.

The relationship between the annealing temperature and the fluorescence change rate (which is positively correlated with the amount of ligated products) closely resembles that of a typical DNA melting assay (see Fig. 3). At low annealing/ligation temperatures (*e.g.* 45–55 °C), the fluorescence signal was constant, suggesting that the probe/target sandwich was stable over this temperature range and hence efficiently ligated. As the temperature was increased further, the signal decreased sharply, implying the dissociation of the probe/target sandwich under such elevated temperatures. The data can be fitted by a sigmoidal function to extract the mimicked *T*_m_ of each DNA sample.^9,10,49^ 
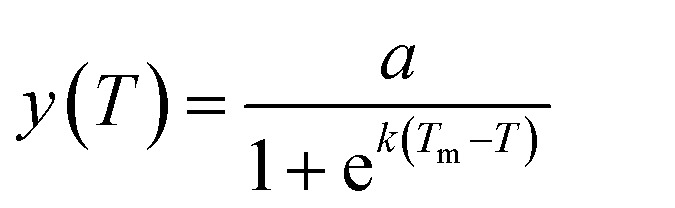


The fluorescence increase rate *versus* ligation temperature can be fitted well by the above equation (with *R* ^2^ = 0.99 and 0.91), yielding *T*_m_ values of 62.6 and 61.1 °C for the pure T2 and 1 : 9 molar mixed T2–T1 targets, respectively (Fig. 3). The presence of a 9-fold excess of the SNP target T1 led to ∼1.5 °C decrease of the effective *T*_m_. This is not unexpected because a fixed amount of total DNA (T1 + T2) was used, so the T2 concentration in the later was only 10% that of the former (T1 could not contribute to positive ligation, see the previous paragraph). The maximum fluorescence response for the (T1 + T2) mixture was ∼15% that of the pure T2 sample, which is likely to be due to the competition of T1 in forming a unligatable probe/T1 duplex, reducing the chances of forming a ligatable probe/T2 duplex. Recently, a colorimetric detection of low abundance mutants with excellent specificity has been reported by Zu *et al.* using the sharp melting behaviour of nanoparticle-immobilised target DNAs.^50^ However, this assay required a stringent temperature adjustment according to the specific target sequences and mutant concentration. In addition, it only gave a nM level of absolute sensitivity. Our approach here, having fM sensitivity together with excellent SNP discrimination ability *via* both the inferred *T*_m_ and fluorescence response, appears to be well-suited for accurate SNP and point-mutation detection.

### Detection of SNPs

**Fig. 4:**
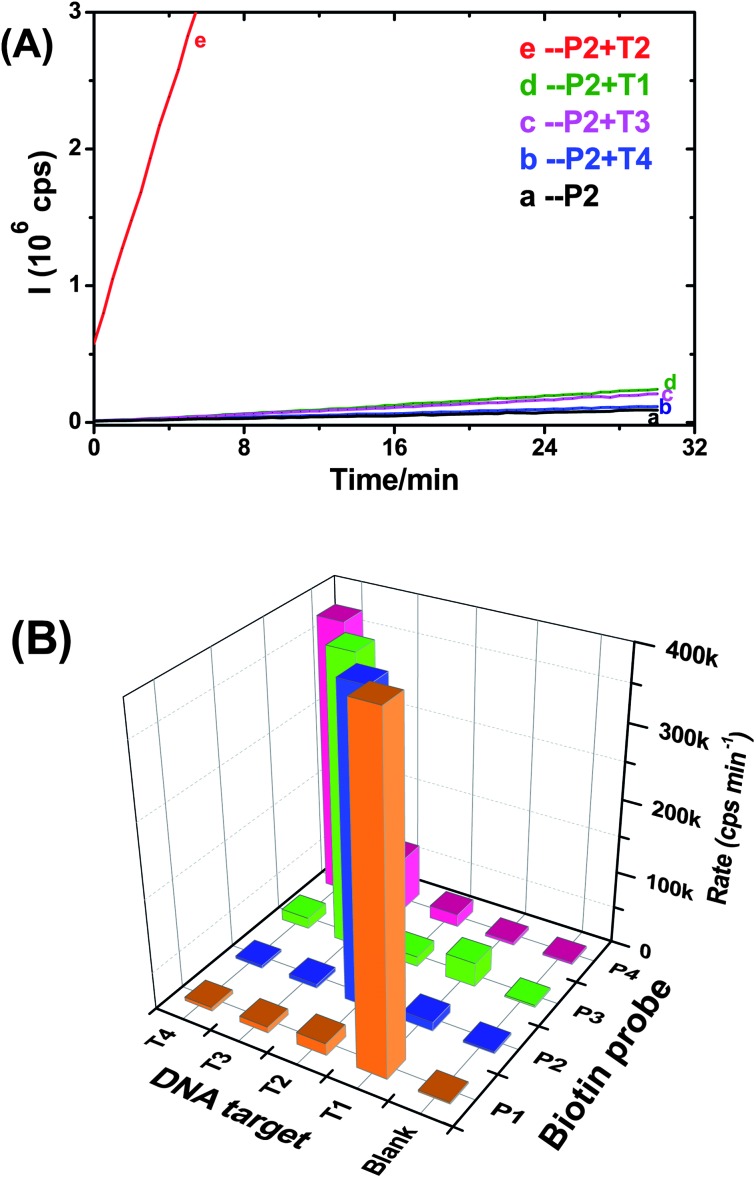
Specificity of the sensor in discriminating the full-match against other single-base mismatch DNA targets. (A) Typical time-dependent fluorescence assay curves of using the P2 (perfect-match to T2) to detect other different SNP targets. (B) Comparison of the average fluorescence increase rates (slopes of fluorescence response curves shown in (A)) for different target/probe combinations (*n* = 3).

We further evaluated the potential of our approach in specific detection of the SNPs in the KRAS gene that are associated with many human cancers (*e.g.* colorectal, pancreas, ductal and lung). In this regard, four DNA targets (100 fmol each) are used in these experiments: the wild-type T1 (counting from 5′, base 17 = C), two cancer mutants: T2 (17C → T), T3 (17C → A), and T4 (17C → G, a cancer irrelevant mutation is used here to demonstrate the general applicability of our sensor), which can be detected by their full-match biotin-probes, P1, P2, P3, and P4, respectively. Typical time-dependent assay fluorescence responses using P2 to detect such DNA targets are shown in Fig. 4A. The full-match P2/T2 clearly exhibited a much greater fluorescence signal than those containing a single-base mismatch, *i.e.* this sensor is highly specific. In fact, this high level of full-match target specificity was achieved throughout the whole probe/target combinations: greatly increased signals were only observed for the full-match probe/target combinations (*e.g.* P1/T1; P2/T2; P3/T3 or P4/T4), but not for those containing a single mismatch (*e.g.* P1/T2–T4; P2/T1, P2/T3–T4; P3/T1–T2, P3/T4 or P4/T1–T3, see Fig. 4B).

Fig. 4 reveals that the fluorescence signals of the later were typically comparable to blank controls, ranging from 0.81 ± 0.09% (for P2/T4) to 4.6 ± 1.0% (P4/T2) that of their respective full-match samples before background correction (BC, except for P3/T1 or P4/T3, which was 8.3 ± 0.9% or 19 ± 4.0% that of the P3/T3 or P4/T4 signal, respectively). The detailed discrimination ratios (DRs) between the full-match P*n*/T*n* (*n* = 1–4) samples over their corresponding single-base mismatch P*n*/T*m* (*n* ≠ *m*; *n*/*m* = 1–4) samples are summarised in Table 2. Impressive SNP DRs of up to 121 (±14) fold before BC (P2/T2 over P2/T4) were obtained, which further increased to up to 383 (±45) fold after BC. This level of SNP discrimination ratio is among the highest reported in the literature (see Table 3). Moreover, even at a relatively low target abundance of 5 fmol, this approach still offered a SNP DR of >3 fold before BC, increasing to 13.5 fold after BC (see ESI, Fig. S3†). All these results confirm the excellent specificity of our approach in SNP detection. It is also noteworthy that all of the perfect-match P*n*/T*n* samples here gave >130 fold greater absolute signals over their respective blank controls, which is considerably higher than most other ultra-sensitive DNA sensors reported recently (see Table 3 for details). For biosensing, a high absolute signal-to-background ratio (SBR) is highly advantageous because not only can this increase the accuracy of target quantification, but also simplify the data analysis by eliminating any requirement of background correction.

**Table 3:** Comparison of the sensing performances of several recently reported, sensitive SNP assays. LOD = limit of detection, where for the full-match DNA/RNA target, this value is given as the total DNA amount and/or concentration; BC = background correction; SNP DR: discrimination ratio between full-match and SNP target; SBR: absolute signal-to-blank background ratio

Sensing method	LOD for target DNA/RNA	SNP DR	SBR	LOD for rare alleles	Reference
Target-recycled ligation + MNP capture + enzymatic amplification	30 amol (0.6 pM)	121 (no BC), 383 (with BC)	130–212	0.75%	This work
Taq DNA polymerase + conjugated polymer FRET	?	∼8	∼8	2%	5
Hybridization reduced quenching of multi-colour molecular beacons immobilised on gold nanoparticles	∼500 pM	∼2.5 to 10	∼5.2 to 30	?	12
Molecular beacon probe + enzymatic amplification	1000 pM (RNA)	∼7	∼10	10%	14
Pyrene excimer probes + hybridization chain reaction	0.26 pM	∼10	∼32	?	15
Endonuclease IV + lambda exonuclease amplification	1 fmol	∼2.2 to 3	∼6	0.5%^*a*^	19
LCR + exonuclease + conjugated polymer FRET	1 fM	∼3	∼6	1%	23
LCR + gold nanoparticle	20 aM	?	?	∼0.1%	24
Nanoparticles-coupled DNA-templated ligation + silver amplification	∼1 pM	∼20 to 30	∼29	?	33
Ligation mediated strand displacement + DNAzyme signal amplification	0.1 fM	106	108	?	42

^*a*^The 0.5% LOD for rare alleles here referred to the SNP over 1,3-double mismatch target, not the full-match target.

### Quantification of rare DNA allele frequency

**Fig. 5:**
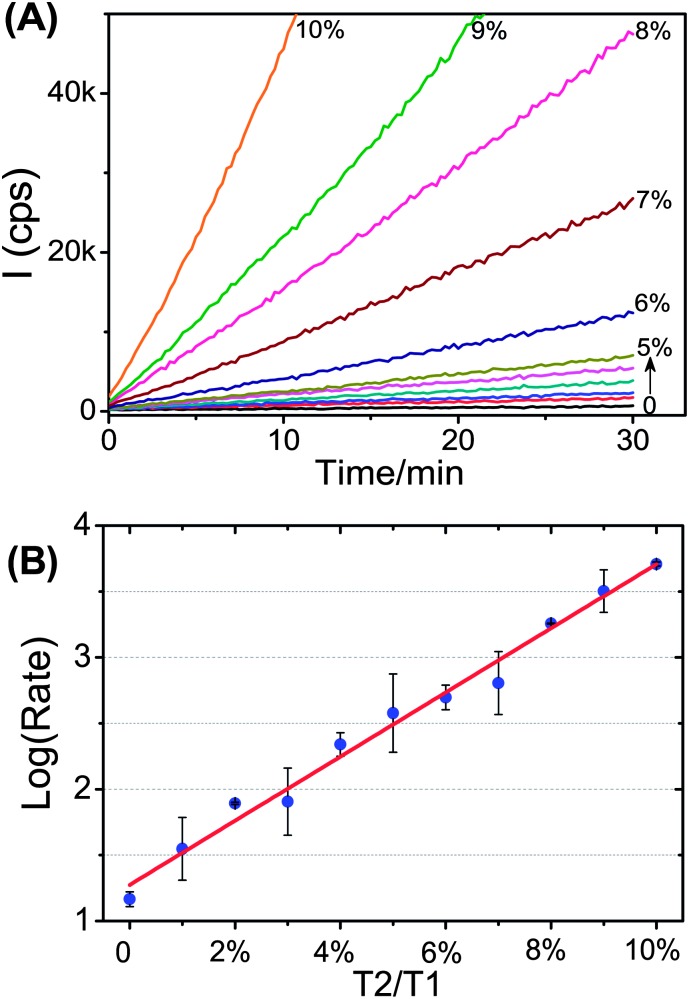
Quantification of the DNA target T2 (KRAS cancer mutant) in the presence of a large excess of the wild type SNP target T1. (A) Time-dependent fluorescence responses of samples with different ratios of T2 to T1, with fixed amounts of T1 (500 fmol). (B) Relationship between the logarithmic fluorescence increase rate and the allele frequency; the data were fitted to a linear function (*R* ^2^ = 0.98).

The high sensitivity and excellent SNP detection specificity make our assay well-suited for quantification of low abundance alleles. To evaluate this potential, a series of samples containing different amounts of the cancer mutant T2 (*e.g.* 0–50 fmol) mixed with a large excess of the wild-type T1 (fixed at 500 fmol) were detected using the P2. The results are shown in Fig. 5.

Fig. 5A reveals that the rate of fluorescence increase initially showed a gradual increase with the increasing T2/T1 (full-match/SNP) ratio of over 0–5%. As the T2/T1 ratio was further increased, the rate of fluorescence response was greatly enhanced: the signal for the 10% T2/T1 sample was a massive 345 fold higher than that for the 0% T2/T1 sample. Moreover, the log (fluorescence increase rate) was found to increase roughly linearly (*R* ^2^ = 0.98) with the increasing T2/T1 ratio (Fig. 5B), where an allele frequency down to as low as 1% was clearly detectable. The estimated theoretical LOD of the allele frequency was 0.75% based on 3*σ*/slope.^48^ Therefore, quantification of the low abundant KRAS 12 cancer mutant (T2) can be realized in >100-fold excess of the wild-type target T1. This result is comparable to or better than most other recently reported, sensitive SNP detection techniques (see Table 3 for details). Furthermore, the detecting limit for low level SNP cancer mutants in a large excess of the coexisting wild-type target here is also comparable to the COLD-PCR/high resolution melting or gene sequencing technique (detection limit: 0.1–1%) developed by the Makrigiorgos group, one of the most sensitive SNP detection techniques.^51,52^ Therefore, this approach appears to be well-placed for sensitive detection of rare point mutations associated with important human diseases (*e.g.* cancer, diabetes, and cardiovascular diseases) as well as studying the SNP–disease correlations.

## Conclusion

In summary, in this paper we have demonstrated a highly sensitive, specific DNA detection approach which combines TRL, strand displacement, MNP mediated target capture/separation, efficient enzymatic signal amplification, and the high efficiency and SNP discrimination ability of the Taq DNA ligase. This approach can quantitate the amol level of unlabelled target DNA with an excellent discrimination ratio, up to 121 fold before BC (increasing to >380 fold after BC), between the full-match and the SNP target. It can quantitate trace amounts of single point cancer mutants in a large excess of wild-type targets with an LOD down to 0.75%. This assay can also mimic DNA melting behaviours, making SNP detection highly reliable. Moreover, it is also general, and can be readily extended to other DNA targets of interest by simply changing the biotin-probes used (*e.g.* P1 for T1; P2 for T2; P3 for T3; P4 for T4 here) and/or other nanoparticle systems. Currently, we are focused on developing more powerful signal amplification strategies to further improve the assay sensitivity and extending its application to real clinical samples.

## Experimental section

### Materials and reagents

Taq DNA ligase and 10× ligation buffer (200 mM Tris–HCl, 250 mM potassium acetate, 100 mM magnesium acetate, 10 mM NAD, 100 mM dithiothreitol and 1.0% Triton X-100, pH 7.6) were purchased from New England Biolabs (UK). All DNA probes and target strands were purchased commercially from IBA GmbH (Germany). They were all HPLC purified by the supplier and their sequences are summarised in Table 1. HRP-NAV and Amplex red were purchased from Thermo Scientific (UK) and Invitrogen Life Technologies (UK), respectively. All other chemicals and reagents were purchased from Sigma-Aldrich (UK) and used without further purification unless otherwise stated. PBS buffer (137 mM NaCl, 10 mM Na_2_HPO_4_, 2.7 mM KCl, 1.8 mM KH_2_PO_4_, pH 7.4) was made with ultra-pure MilliQ water (resistance > 18 MΩ cm^–1^). The MNPs were in house synthesized and modified (see ESI† for details).

### Experimental methods

TRL reaction was performed in 50 μL 1× ligation buffer containing the Taq DNA ligase (25 units), biotin-probe and phosphate-probe (1 pmol each), and the various concentrations of mutant or/and wild-type DNA targets. 30 thermal cycles were carried out, with each cycle consisting of a 2 min denaturation at 95 °C and 5 min annealing/ligation at 45 °C. After the final cycle, the Taq ligase was inactivated by addition of EDTA (8 μL, 50 mM). Subsequently, the capture-DNA modified MNPs (20 μg, loaded with ∼10 pmol of the Cap-DNA) were added to initiate strand displacement and ligated product capture at room temperature overnight, although incubation for 1 hour was found just as effective as incubation overnight. After magnetic separation, the MNPs with bound ligated products were dispersed in 200 μL of buffer A (PBS + 1 mg per mL BSA) and incubated with HRP-NAV (1 pmol) for 0.5 h. The MNPs were then magnetically separated (assisted with a brief ∼30 s centrifugation) and then washed once with PBS, twice with buffer B (PBS + 0.1% Tween-20) and once more with PBS. The MNPs (with bound enzymes) were subsequently transferred to a 96-well plate for high throughput screening where different sensing conditions were evaluated simultaneously. The enzymatic assay was triggered by the addition of Amplex red and H_2_O_2_ (2 μM each, final concentration) in PBS (200 μL of the final assay volume, pH 7.40), and real-time fluorescence changes were monitored on an Envision plate reader using BODIPY TMR FP 531 as an excitation filter and Cy3 595 as an emission filter.

For mimicking the DNA melting assay, the experimental procedures used were similar to what were described above, except that only five thermal cycles were used. Each cycle included a 2 min denaturation at 95 °C and a 5 min annealing/ligation at specific temperatures shown in Fig. 3.
