# Supplementary material for: Ultrasensitive single-nucleotide polymorphism detection using target-recycled ligation, strand displacement and enzymatic amplification
Source: Nanoscale. 2013 May 2;5(11):5027–35. doi: 10.1039/c3nr01010d (PMC4576341; doi:10.1039/c3nr01010d)
Supplement: Supplementary file 1 [file NR-005-C3NR01010D-s001.pdf]

Electronic Supplementary Material (ESI) for Nanoscale
This journal is © The Royal Society of Chemistry 2013

Electronic Supporting Information (ESI)

Ultrasensitive single-nucleotide polymorphism detection using target-recycled ligation, strand displacement and enzymatic amplification

Yue Zhang,[a] Yuan Guo,[a,*] Philip Quirke[b] and Dejian Zhou[a,*]

[a] *School of Chemistry and Astbury Centre for Structural Molecular Biology, University of Leeds, Leeds LS2 9JT, United Kingdom. Fax:+44-113-3436565; E-mail: y.guo@leeds.ac.uk (YG) and d.zhou@leeds.ac.uk (DZ).*

[b] *Section of Pathology and Tumour Biology, Leeds Institute of Molecular Medicine, Wellcome Trust Brenner Building, St James's University Hospital, University of Leeds, Leeds LS9 7TF, United Kingdom.*

A) *Preparation and modification of the MNPs*

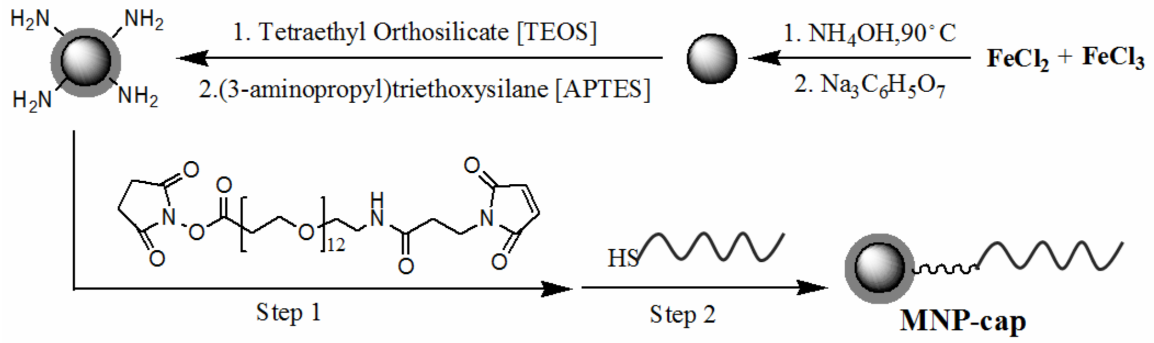

Scheme S1. Illustration of the procedures for MNPs Preparation and modification. It should be noted that several hundred strands of the capture-DNAs are attached to each MNP, and only one is shown here for simplicity.

1) *Preparation of citrate-Modified Fe_3O_4 MNPs*

5 mmol $FeCl_2{\cdot}4H_2O$ and 10 mmol $FeCl_3{\cdot}6H_2O$ were dissolved in 50 mL deoxygenated H_2O under magnetic stirring. Then 4.3 ml $NH_3{\cdot}H_2O$ (35%) solution mixed with 50 mL deoxygenated H_2O was added into the above solution dropwisely with N_2 gas bubbling. The mixture was heated at 80 ^oC for 30 min in an oil bath under vigorous stirring and then for 2 h at 90 ^oC following the addition of 50 mL of 0.2 M trisodium citrate (see Scheme S1). The obtained Fe_3O_4 magnetic nanoparticles (MNPs) appeared as black precipitate which was separated by centrifugation (4000 g for 3 min) and then washed once with acetone. Finally, the MNPs were dispersed in deionized water (with the volume being adjusted to 30 mL).[1-4]

2) *Preparation of Fe_3O_4/SiO_2 core/shell MNPs*

Fe_3O_4/SiO_2 core/shell MNPs were prepared by using a modified Stöber method.[5] In typical preparation, 1 mL of the hydrophilic Fe_3O_4 seeds prepared above was firstly diluted with the mixture of 4 mL deionized water and 20 mL absolute ethanol. Under continuous stirring and N_2 bubbling, 0.43 mL $NH_3{\cdot}H_2O$ (35%) solution and 0.5 mL tetraethyl orthosilicate (TEOS) were consecutively added to the above suspension. The hydrolysis/condensation reaction was

allowed to proceed for 3 h under N_2 gas bubbling at room temperature (see Scheme S1). Subsequently the obtained Fe_3O_4/SiO_2 core/shell MNPs were separated by centrifuging at 4000 rpm for 5 min and then washed by water twice. Finally, the obtained MNPs were re-dispersed in deionized water (total volume:1 mL).

3) Preparation of NH_2-Functionalised MNPs (MNP-NH_2)

Amine functionalised Fe_3O_4/SiO_2 core/shell MNPs were prepared as follow: [2, 4-8] 1 mL of the above silica-coated Fe_3O_4/SiO_2 core/shell MNP prepared above was dispersed in 4 mL deionized water and 20 mL absolute ethanol. Then 0.43 mL $NH_3{\cdot}H_2O$ (35%) solution and 0.5 mL of (3-aminopropyl)triethoxysilane (APTES) were consecutively added to the solution under continuous stirring and N_2 gas bubbling. The reaction was carried out for 3 h at room temperature, leading to APTES to hydrolyse and subsequently condensed on the MNP surface to introduce amine groups (see Scheme S1). Afterwards, the amine functionalized MNPs (NH_2-MNPs) were separated, washed and then re-dispersed in deionized water. Concentration of the MNP suspension was estimated by comparing the suspension's weight with pure water of the same volume.

4) Preparation of Capture-DNA immobilized MNP (Cap-MNP)

5 mg NH_2-MNP and 2.5 mg SM(PEG)$_{12}$ were mixed and incubated in Buffer C (PBS buffer containing 1mM EDTA, pH = 7.2) at room temperature for 1 h, leading to the MNP surface being functionalised with maleimide groups. Thereafter, the MNPs were washed by Buffer C twice, and then 5 nmol of thiolated capture-DNA in 1 mL Buffer C was added into the above MNPs and incubated for 1 h at room temperature. The capture-DNA is covalently conjugated to the MNP surface *via* Michael addition of the thiolate to the MNP surface maleimide groups (see Scheme S1). The MNPs were subsequently washed twice by Buffer C. It should be noted that all of the original and washing supernatants were collected and combined for UV measurement at 260 nm to determine the amount of free-unbound capture-DNA, allowing the estimation of the capture-DNA conjugation efficiency on the MNP. The capture-DNA loaded MNPs were then treated with 5 μL of 2-mercaptoethanol in 1 mL Buffer A (PBS plus 1 mg/mL BSA) to cap any unreacted maleimide groups and to block the MNP surface to reduce non-specific absorption of HRP-NAV. Typically, the capture-DNA loading on the MNP is ~0.4-0.5 nmol/mg of MNP.

B) Magnetic retrieving property of the MNP-NH_2

Fig. S1 shows that MNPs are well-dispersed in water, forming a uniform suspension before applying an external magnetic field. Upon placing onto a standard biomag separation device for 1 min, almost all of the MNPs have been attracted to the side wall of the tube, confirming that the MNPs are easily retrieved by applying a magnetic field for easy magnetic separation.

A B

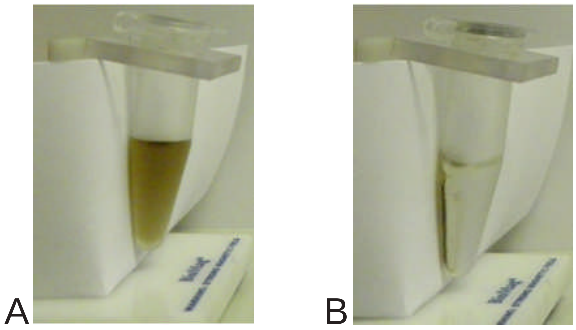

Fig. S1. Representative photographs of the MNP-NH_2 dispersed in water on being placed onto a biomag separation device (**A**) and after waited for 1 min (**B**).

C) Optimization of the Cap-MNP probe and thermal cycle numbers

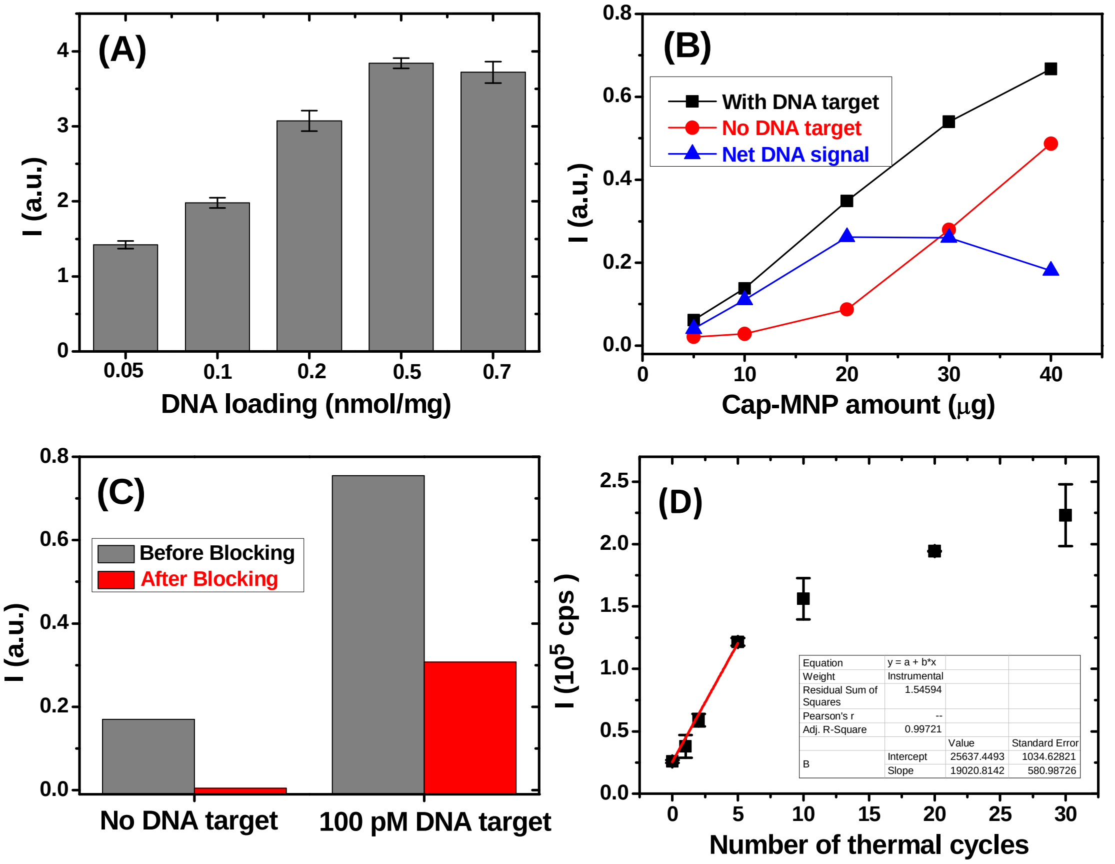

Fig. S2. Optimisation of the Cap-MNP probes and assay procedures.

(A) Effect of capture-DNA loading on the MNP on the capture efficiency (assay fluorescence response) for 5 fmol target DNA. The signal initially increases with the increasing Cap-DNA loading on the MNP, but saturates at ~0.5 nmol/mg. Therefore this loading is determined as optimum, and is used for all subsequent assays.

(B) Effect of the Cap-MNP amount on the signal response for detecting 5 fmol DNA target: **red dots** show the assay without the DNA target (background due to non-specific adsorption), and **black squares** show the assay with 5 fmol DNA target, and **blue triangles** (**black squares** - **red dots**) show the net contribution from the DNA target. 20 μg Cap-MNP is found to be optimum here because it gives the maximum net signal from the DNA target.

(C) Effect of Cap-MNP surface blocking (by 2-mercaptoethanol and 1 mg/mL BSA, see MNP preparation part) on the assay specificity for DNA target detection. The grey and **red** bars show the assay prior to and after the blocking, respectively. The signal/background ratio for 5 fmol DNA target increases from ~4.4 (before blocking) to ~62 after blocking, confirming surface blocking is essential to achieve high signal/background ratio. Therefore all Cap-MNPs have been blocked by 2-mercaptoethanol and BSA before being used.

(D) Effect of thermal cycle number on the rate of fluorescence increase. The signal increases linearly during the first 5 cycles (indicating linear amplification), but the rate of increase gradually levels off as more thermal cycles are used (due to depletion of the signal DNA, a 10 fold signal DNA to target DNA ratio is used here). Nevertheless, the assay with 30 thermal cycles gives the highest signal, being 8.6 times that without thermal cycle, confirming the success of the TRL (target recycled ligation) for signal amplification. 30

thermal cycles are therefore used in all assays except for mimicking the DNA melting experiments (Fig. 3) and the work described in Fig. S3 below where only 5 thermal cycles are used.

D) Discrimination of SNPs at low target abundance

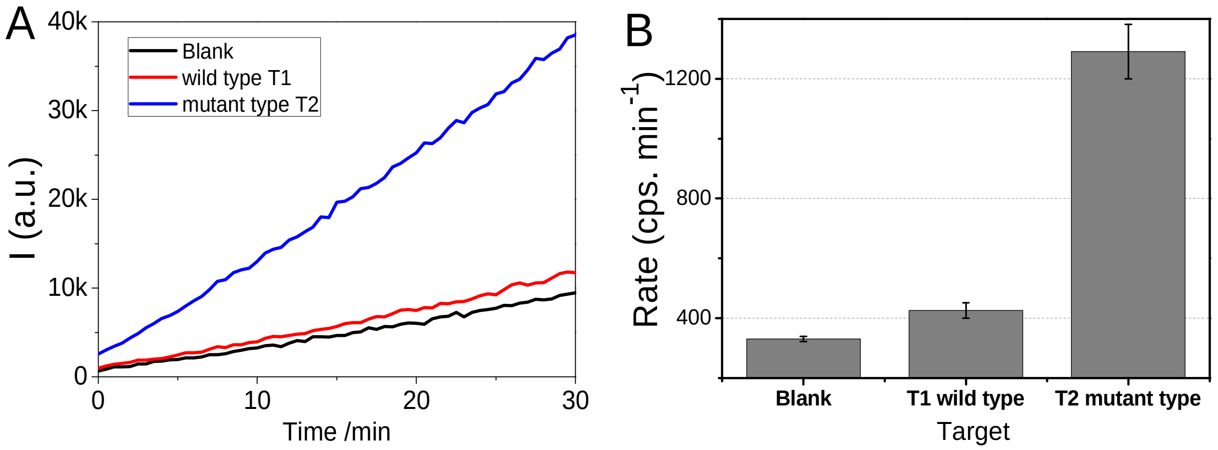

Fig. S3. Discrimination between the full-match (T2) and single mismatch target (wild-type T1) at low target abundance (5 fmol) by our assay: five thermal cycles with an annealing temperature of 45 °C were used. (**A**) Time-dependent fluorescence intensity change for the blank control (**black**), SNP target T1 (**red**) and perfect-match target T2 (**blue**). (**B**) The corresponding fluorescence rate changes of the three samples.

The discrimination ratio between the full-match T2 and the SNP target T1 was determined as ~3.03 folds before background correction, which was increased to ~13.5 folds after background correction (*e.g.* Blank control background signal was subtracted from the T2 and T1 signals).

References

1. C. Hui, C. M. Shen, T. Z. Yang, L. H. Bao, J. F. Tian, H. Ding, C. Li and H. J. Gao, *J. Phys. Chem. C*, 2008, **112**, 11336-11339.
2. Z. L. Lei, Y. L. Li and X. Y. Wei, *J. Solid State Chem.*, 2008, **181**, 480-486.
3. Z. Lei, X. Pang, N. Li, L. Lin and Y. Li, *J. Mater. Process. Technol.*, 2009, **209**, 3218-3225.
4. Y.-F. Huang, Y.-F. Wang and X.-P. Yan, *Environ. Sci. Technol.*, 2010, **44**, 7908-7913.
5. W. Stober, A. Fink and E. Bohn, *J. Colloid Interface Sci.*, 1968, **26**, 62-&.
6. E. P. Plueddemann, ed., *Silane coupling agents*, Plenum Press, New York, 1991.
7. M. Ohmori and E. Matijevic, *J. Colloid Interface Sci.*, 1993, **160**, 288-292.
8. I. J. Bruce and T. Sen, *Langmuir*, 2005, **21**, 7029-7035.
